# Predicting health, economy, and environmental indicators: a comparative machine learning approach

**DOI:** 10.1186/s12889-026-28472-0

**Published:** 2026-07-22

**Authors:** Murat Tekbaş, Elif Aktepe

**Affiliations:** 1 Afyon Vocational School, Management and Organization Department, Afyon Kocatepe University, Afyonkarahisar, 03200 Turkey; 2 Afyon Vocational School, Electronics and Automation Department, Afyon Kocatepe University, Afyonkarahisar, 03200 Turkey

**Keywords:** Machine learning, Economic indicators, Environmental indicators, Health expenditure, Ensemble learning

## Abstract

**Background:**

This study aims to reveal the interaction between environmental sustainability and health policies by examining the relationships between economic growth, carbon emissions, and healthcare expenditures within the framework of machine learning methods. The primary motivation of the research is to evaluate the relationships among economic growth, health indicators, and environmental sustainability using data-driven methods.

**Methods:**

The analysis is based on a large-scale panel dataset covering 217 countries for the period 1960–2024, obtained from the World Bank World Development Indicators database. Advanced machine learning algorithms, including Extra Trees, Random Forest, Extreme Gradient Boosting, Histogram-based Gradient Boosting, and Light Gradient Boosting Machine, were implemented, and their prediction performances were compared. To improve prediction accuracy, a hybrid ensemble model was proposed by combining the highest-performing algorithms. For model interpretability, the relative importance levels of variables were analyzed using the Explainable Artificial Intelligence approach via Shapley Additive Explanations.

**Results:**

The findings indicate that the proposed hybrid ensemble model provides higher prediction accuracy compared to individual algorithms. Results from the Shapley Additive Explanations analysis show that health expenditures, economic growth, and innovation-related indicators exhibited high predictive importance within the developed machine learning models. The findings highlight the predictive associations between environmental indicators, healthcare spending, and sustainable development outcomes.

**Conclusions:**

This study analyzes the complex relationships between economic growth, environmental sustainability, and health policies through a data-driven perspective. The findings provide additional evidence regarding the predictive associations among economic, environmental, and health-related indicators. The proposed framework demonstrates the usefulness of machine learning approaches for analyzing complex nonlinear patterns in large-scale country-year datasets.

## Background

Economic growth is one of the most fundamental macroeconomic indicators determining a country’s welfare level, production capacity, and development performance. Traditional growth theories explain economic growth through basic factors of production such as capital accumulation, labor, and technological progress. However, the modern growth literature shows that economic performance cannot be explained solely by these classical factors, and that health investments, technological innovation, and various macroeconomic variables also play significant roles in the dynamics of economic growth. In particular, human capital and technological development are considered key predictors of sustainable economic growth.In recent years, the relationships between economic growth and environmental indicators have also gained increasing importance in the literature. Increased economic activity can drive higher production and energy consumption, leading to rising carbon emissions. Therefore, the relationships between economic growth and carbon emissions have become an important research area in the sustainable development literature [1, 2]. Although energy consumption is widely used in the related literature, it was not included in the present analysis due to data availability constraints and the aim of maintaining model interpretability.

In this context, the relationship between health expenditures and environmental pollution is primarily addressed in the literature within the framework of ‘pollution-related costs. Empirical findings show that as air pollution increases, health problems become more widespread, leading to a significant increase in healthcare expenditures [3]. The positive relationship between toxic emissions, particularly carbon dioxide (CO₂) and NOX, and healthcare costs is a significant financial pressure on public budgets, hindering sustainability [4, 5]. In this context, it is emphasized that air pollution negatively affects both public health and macroeconomic performance, and that increased healthcare costs suppress growth by diverting economic resources to inefficient uses [5]. Therefore, health expenditures have become an important indicator associated with macroeconomic and environmental conditions.

The methods used to analyze macroeconomic and environmental indicators have diversified significantly in recent years. While traditional econometric models often rely on linear assumptions, machine learning (ML) algorithms are increasingly used in economic analysis due to their capacity to analyze high-dimensional datasets and reveal nonlinear relationships [6]. In this context, many studies show that ML methods can achieve higher prediction accuracy in economic growth forecasting than traditional methods [7–9]. Similarly, it has been shown that ML algorithms perform well at predicting carbon emissions and analyzing their determinants [10, 11].

A review of the current literature reveals two significant gaps. Firstly, a large portion of the ML literature focuses separately on predicting economic growth or on the determinants of carbon emissions. Studies that analyze the combined effects of health investments, innovation indicators, and macroeconomic variables on both economic growth and carbon emissions are quite limited. Secondly, a significant portion of existing studies focus on a single country or specific country groups, and the number of studies using large global datasets is limited.

This study, informed by gaps in the literature, aims to analyze the complex and dynamic relationships among health expenditures, carbon emissions, and Gross Domestic Product (GDP) from a ML perspective. The study uses a 10-variable dataset comprising economic indicators, social variables, and technological parameters. Methodologically, the research is based on a workflow that begins with data normalization using StandardScaler and the removal of missing values, followed by allocating 80% of the data for training and 20% for testing. In the estimation process, advanced algorithms, including Random Forest (RF), Extreme Gradient Boosting (XGB), and the Hybrid Model (ET + HGB + XGB), were tested alongside classical regression models. Furthermore, the relative predictive contributions of the variables were analyzed using explanatory artificial intelligence (XAI) methods.

In this context, the study contributes to the literature in three ways. Firstly, the relationships among economic growth, carbon emissions, and health expenditures are analyzed from a ML perspective using a large-country sample and a long-term dataset. Secondly, the most effective methods for predicting sustainable development indicators are determined by comparing the performance of different ML algorithms. Thirdly, the relative importance of variables is revealed through the proposed hybrid model and XAI approach, and new empirical findings are presented regarding predictive relationships among economic growth, environmental sustainability, and health-related indicators.

### Theoretical framework

The determinants of economic growth have long been one of the central research areas in economics. Traditional growth theories explain economic growth through fundamental production factors, including capital accumulation, labor, and technological progress.. However, modern growth literature emphasizes that health investments, technological innovation, and various macroeconomic dynamics also play important roles in shaping economic performance [12, 13]. In addition, economic growth processes are closely associated with environmental outcomes such as energy consumption, environmental degradation, and carbon emissions. Therefore, the theoretical framework of this study is based on the multidimensional relationships among economic growth, health, innovation, and environmental sustainability.

#### Theoretical framework of economic growth: health, innovation, and macroeconomic dynamics

In the literature, health indicators are considered an important component of human capital. It is widely accepted that healthier individuals increase productivity and support economic performance [14, 15]. Therefore, health expenditures are regarded as important indicators associated with economic growth. Similarly, technological innovation and knowledge production are widely associated with long-term economic growth [16, 17]. Indicators such as patent counts, R&D measures, and innovation capacity are widely used to reflect countries’ technological development levels and competitiveness [18].

In addition, various macroeconomic variables such as trade openness, foreign direct investment, and natural resource revenues are considered important factors affecting economic growth [19]. However, these relationships may vary depending on institutional structure, financial development, and production characteristics.Therefore, economic growth should be evaluated not only through traditional macroeconomic indicators, but also together with health, innovation, and environmental factors.

#### Theoretical framework of economic growth and carbon emissions

The relationship between economic growth and environmental indicators constitutes one of the main research areas in the sustainable development literature. In this context, the Environmental Kuznets Curve (EKC) hypothesis suggests that environmental degradation may increase during the early stages of economic growth, but environmental performance may improve beyond a certain income level through technological progress and environmental policies [20, 21]. The literature also emphasizes that energy-intensive production structures, technological development, and environmental policies in shaping the relationship between economic growth and carbon emissions [22, 23]. In addition, innovation and renewable energy technologies are considered important factors supporting environmental sustainability [24]. However, the relationship between economic growth and carbon emissions may differ across countries depending on institutional structures, energy policies, and sustainable development strategies.

### Literature review

Studies examining the determinants of economic growth and carbon emissions have increased significantly in recent years. The widespread use of large datasets and advances in computational capacity have accelerated the application of ML methods to the analysis of economic and environmental indicators. Accordingly, studies examining the prediction and determinants of economic growth using ML methods, and studies analyzing the prediction of carbon emissions and factors related to environmental sustainability, are prominent in the literature. This section examines the relevant literature under two main headings.

#### Analysis of economic growth with ML in the context of macroeconomic and health indicators: literature review

Recent studies increasingly employ machine learning algorithms to predict economic growth because of their ability to model nonlinear relationships and improve predictive performance. As summarized in Table 1, these studies generally focus on algorithmic accuracy and country-specific applications. However, relatively few studies jointly consider health expenditures, innovation indicators, and environmental variables within a global-scale predictive framework.

**Table 1:** Selected studies on predicting economic growth using ML methods

Author	Period	Sample	Method	Variables	Key Findings
Stock and Watson (2012) [25]	1960Q2—2008Q4	USA	Shrinkage methods	Economic Growth, Employment, Unemployment, Inflation	Using macroeconomic indicators together increases the accuracy of economic growth forecasts.
Bajari et al., (2015) [6]	2008	USA	RF, SVR	Demand indicators	Income and consumption indicators were found to be significant predictors of economic growth.
Yoon, J (2021) [9]	2001 −2018	Japan	RF, GB	Economic growth, Industrial production index, FDI	Industrial production and investment indicators were found to have a positive effect on economic growth.
Claveria et al., (2021) [7]	2003Q1–2020Q1	19 European Countries	ML-based models	Sentiment indicators	Consumer confidence and economic expectations were found to strongly explain GDP growth.
Lee and Msefula (2024) [26]	1984Q1-2020Q2	Taiwan	SVM	Macroeconomic indicators	Trade volume and investment indicators were found to positively affect economic growth.
Yang et al., (2024) [8]	1992Q1-2023Q4	China	ML models	Economic growth, Construction sector, Production, Investment, Export and Import	Industrial production, trade, and investment indicators were identified as key drivers of economic growth.
Passarella et al., (2025) [27]	1970 −2023	Indonesia	RF, SVR, XGB	Economic Growth, FDI, Export, Import	Trade openness and investment indicators were found to have strong effects on economic growth.
Jönsson K (2026) [28]	1996Q1–2024Q2	Sweden	RF, GB	Economic growth, Production, Investment, Trade, Service sector	Business expectations and production indicators were found to strongly explain GDP growth.
Fotopoulou et al., (2026) [29]	1997—2023	Venezuela	RF	Satellite data, Macro indicators	Nighttime lights data was found to be a strong predictor of economic activity and GDP growth.

The studies summarized in Table 1 demonstrate that machine learning methods provide successful results in predicting economic growth. However, the existing literature mainly focuses on specific country cases, limited variable sets, and model prediction performance. In particular, studies jointly examining health expenditures, environmental indicators, and innovation variables within a unified analytical framework remain limited. Moreover, many existing studies do not incorporate XAI techniques to improve the interpretability of model outcomes. This limitation restricts a more comprehensive understanding of the multidimensional relationships among economic growth, environmental sustainability, and health policies.

#### Analysis of carbon emissions with ML in the context of macroeconomic and health indicators: literature review

The use of ML methods in predicting carbon emissions has also become increasingly widespread in recent years. Many studies in the literature use machine learning algorithms to analyze the relationships between economic growth, energy consumption, urbanization, trade openness, technological development, and carbon emissions. A significant portion of these studies demonstrates that ML models achieve higher prediction accuracy in carbon emission forecasting than traditional econometric methods. In particular, algorithms such as RF, ANN, SVM, and GB are frequently preferred for analyzing environmental indicators. Studies analyzing carbon emissions using ML methods are presented in Table 2.

**Table 2:** Selected studies on the analysis of carbon emissions with ML methods

Author(s)	Period	Sample	Method	Variables	Key Findings
Mardani et al., (2020) [30]	1962—2016	Panel	ANN, SOM	Energy consumption, economic growth	It has been determined that ML models provide high accuracy in carbon emission prediction.
Li et al., (2022) [31]	2000—2018	China	ML algorithms	Economic Growth, Energy consumption, R&D investments, urbanization, FDI	Energy consumption and industrialization were found to be the most important determinants of carbon emissions.
Ahmed et al., (2022) [32, 33]	1990—2014	China & India	Long Short-Term Memory (LSTM	Economic growth, Financial Development, Energy consumption	Energy consumption is one of the factors that most strongly increases carbon emissions.
Zhao et al., (2022) [34]	2000—2019	China	RF, SVR	Energy consumption, economic growth	It was concluded that economic growth and population have an impact on carbon emissions.
Chang et al., (2023) [35]	1970—2021	China	Ensemble ML	Economic indicators	Ensemble learning methods provide higher accuracy in carbon emission estimation.
Li and Zhang (2023) [36]	2021M1—2022M10	China	ML models	Energy consumption, Industrial production, Logistics	Industrial activities and energy use are the main determinants of carbon emissions.
Wang & Yu (2023) [37]	2000—2018	China	SVR, RF	Fossil fuel consumption, Gas consumption	It is stated that fossil fuel consumption significantly affects carbon emissions negatively (increases them).
Xiang et al., (2023) [38]	2010—2019	China	ML algorithms	Local Fiscal Policies, Land use, energy consumption	Variables showed a tendency to concentrate carbon emissions in parallel with the increase in sample value.
Chen et al., (2024) [39]	1990—2019	India	ML models	Digital technology, entrepreneurship, economic growth	Economic growth and technological developments can increase carbon emissions through the energy consumption channel.
Cooray and Özmen (2024) [40]	2002—2020	22 European Union Countries	ML methods	Population, Economic growth and technology, Institutional quality	It is detected that population density, urbanization, and energy consumption cause more carbon emissions than institutional governance.
Guo et al., (2024) [41]	1995—2022	China	ML algorithms	Transportation Sector	Renewable energy consumption and tightening of fuel emission standards are seen as important for carbon emissions.
Huang et al., (2024) [42]	2012—2021	China	ML models	Economic growth, urbanization, Building Climate Classification	Building climate classification and electrification rate are determinants of carbon emissions.
Xing et al., (2024) [43]	2006—2020	China—284 Cities	ML Methods	Energy consumption, economic growth, population	Energy consumption, economic growth, and population significantly affect carbon emission efficiency.
Yao and Li (2024) [44]	1978—2016	China	RF	Energy consumption, economic growth, technology	It was stated that the contribution of technological innovation to reducing carbon emissions is high.
Ye et al., (2024) [45]	1993—2021	China	ML Algorithms	Industrial production, technological innovation	It is assessed that the transformation of the industrial structure and the provision of technological innovation are necessary.
Zhang et al., (2024) [46]	2024	China—282 Cities	ML Models	Inspection Policy	It was determined that environmental policies increase carbon emission efficiency.
Dai et al., (2025) [47]	1980Q1—2021Q2	China	Ensemble ML	Energy consumption, economic activity	Ensemble learning methods provide the highest accuracy in carbon emission forecasting.
Dong et al., (2025) [48]	2002—2017	China	Explainable ML	Population, economic growth, knowledge complexity	Population density, economic growth, and knowledge complexity increase carbon emission intensity.
Hong et al., (2025) [49]	1997—2021	China	SVR, ML models	Energy consumption, technology	Encouraging low-carbon technology and transforming the energy structure are seen as important in reducing carbon emissions.
Wu et al., (2025) [50]	2005—2021	China – 41 Cities	ML algorithms	Building Carbon Emissions, energy consumption	Urbanization and energy consumption increase residential emissions.
Xie et al., (2025) [51]	2010—2019	China – 667 Cities	ML models	Continuous shrinkage of cities, smart shrinkage, continuous growth	Urban growth has an increasing effect on carbon emission intensity.
Krishnendu and Patra (2025) [1]	1980—2022	20 Countries	ML + STIRPAT	Economic growth, trade openness, quality of government institutions	Growth in low-income countries negatively affects the environment. In wealthy countries, the impact on the environment is decreasing.
Magazzino & Mele (2025) [2]	1970—2017	Russia	ML algorithm	Energy consumption, economic growth	Energy consumption and economic growth increase carbon emissions.
Xiong et al., (2025) [52]	2000—2022	152 Countries	ML models	Foreign aid	There is a non-linear relationship between foreign aid and carbon emissions.
Baydar & Mete (2026) [53]	2007—2022	38 OECD countries	ML algorithms	Logistics performance, economic growth	Economic growth makes a high contribution to carbon emissions, while logistics performance and trade openness make a low contribution.

When examining the studies presented in Table 2, it is evident that machine learning algorithms have been widely used in recent years to predict carbon emissions. In particular, algorithms such as Random Forest, XGBoost, and Gradient Boosting have demonstrated strong performance in modeling nonlinear relationships between environmental and economic indicators. However, a substantial portion of the existing studies focuses on specific country cases or limited environmental variables. Studies jointly examining health expenditures, economic growth, and carbon emissions within the same analytical framework remain highly limited.

#### Analysis of health indicators with ML: literature review

There are numerous studies in the literature examining the interaction between health expenditures and environmental degradation. A significant portion of these studies reports that economic growth and increasing energy consumption are associated with lower environmental quality and higher financial pressure on health systems [31]. While health expenditures are generally considered a “compensation cost” for environmental pollution in the traditional literature, current research has also raised the possibility that they could serve as a “driving force” in reducing emissions [54]. Analyses conducted particularly BRICS countries report dynamic and sometimes nonlinear, relationship between health investments and environmental sustainability goals, using various econometric methods [55]. However, the scope of the issue has expanded in recent years to include the interaction between demographic shifts and energy budgets. Previous studies have emphasized the complex relationships between health expenditures and environmental pressures within the broader framework of sustainable development.

Furthermore, modeling the positive impact of climate change mitigation policies on air quality through “co-benefits” in healthcare costs has brought the issue into a more holistic energy-health framework [56]. Unlike traditithis onal literature, recent studies have attempted to redefine the relationship between healthcare expenditures and environmental indicators using artificial intelligence and ML algorithms. These few new-generation studies reveal complex nonlinear relationships between variables while also focusing on the environmental costs of the digitalization process of healthcare systems.

In light of the current literature summarized in Table 3, the majority of studies examining the interaction between health expenditures and environmental indicators still rely on traditional econometric models. A review of the existing literature indicates that studies jointly addressing health expenditures, environmental indicators, and economic performance remain highly limited. In particular, most studies focus either solely on carbon emissions or exclusively on economic growth prediction. Furthermore, a considerable portion of the existing research is restricted to small sample groups or single-country analyses. This situation limits a comprehensive understanding of multidimensional and nonlinear relationships at the global level and increases the need for more integrated methodological frameworks supported by explainable artificial intelligence techniques.

**Table 3:** Selected studies on predicting health indicators using ML methods

Author(s) and Year	Scope/Dataset	Methodology	Key Findings/Focus
Richie (2022) [57]	AI Use in Healthcare	Ethics and Sustainability Analysis	While the goals of medicine are ethically supported using health and resource conservation criteria, the necessity of integrating fair sustainability into healthcare services is emphasized.
Das and Chandra (2023) [58]	Healthcare Services	Literature Review (Survey)	AI and ML applications in the healthcare sector enable the reduction of emissions through data-driven optimization and the achievement of ‘Green AI’ goals.
Abdelsamiea et al. (2026) [59]	Global Data	GB	It is stated that the population over 65 is the strong predictor of health expenditures (93%) and there is a strong positive relationship between CO₂ emissions and health expenditures.
Caravaggio et al. (2026) [60]	Panel Data—21 Regions of Italy	GB Machine	ML results show that health expenditures are influenced by economic, demographic, and environmental factors, and CO₂ emissions are a important predictive factor.

## Methods

**Fig. 1:**
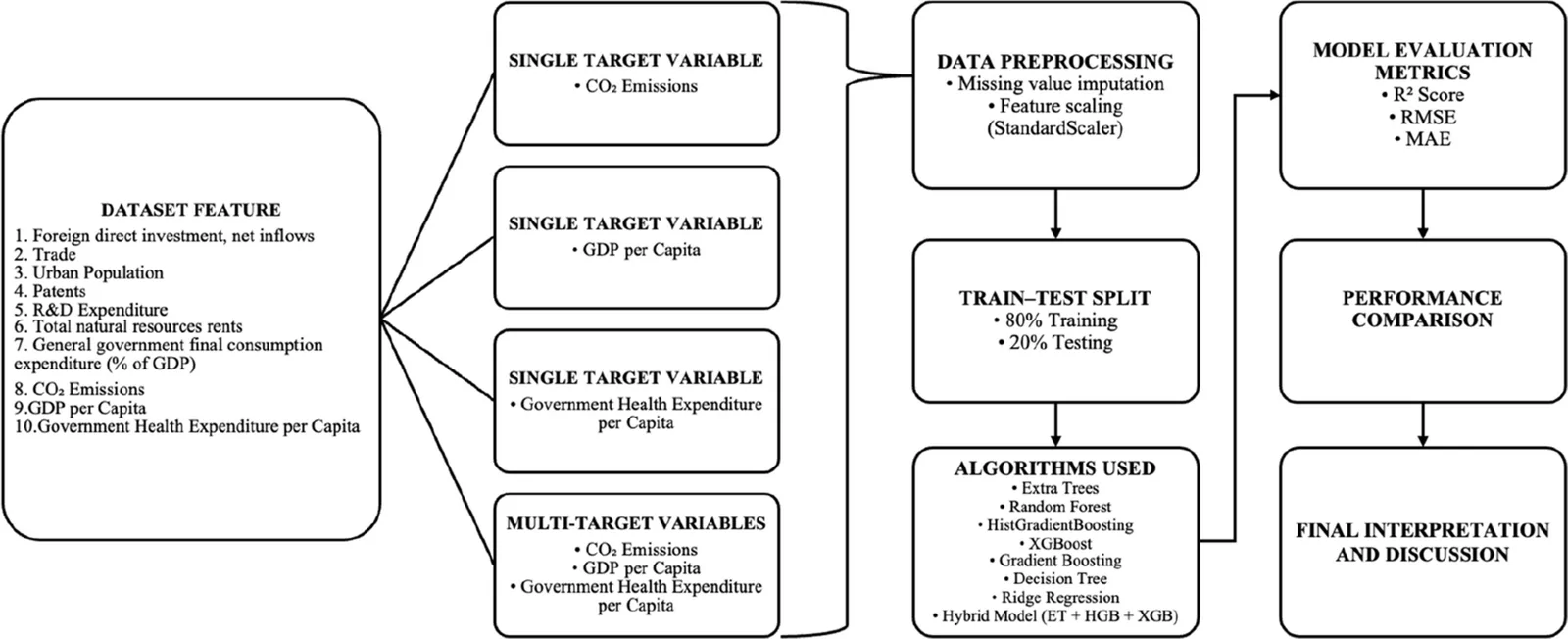
Proposed machine learning workflow used in the study, including data preprocessing, train–test splitting, model development, performance evaluation, and interpretation stages

This study proposes a machine learning framework based on advanced algorithms and hybrid ensemble models to predict carbon emissions, GDP, and health expenditures using economic, demographic, and technological indicators under single- and multiple-output scenarios. The proposed ML framework is presented in Fig. 1. This structure consists of a multi-stage analysis process encompassing data preprocessing, model training, and performance evaluation, starting with the definition of dataset features. In the first stage, the dataset containing economic, environmental, and technological indicators was defined as input to the model. Then, a data preprocessing step was applied, including the removal of country-year observations containing missing values in the selected variables and feature scaling. After the data-cleaning procedure, the final analytical dataset contained no missing values. During the modeling process, four prediction scenarios were created: three single-output prediction scenarios focusing separately on CO₂ emissions, GDP, and health expenditure, as well as one multiple-output scenario in which these variables were predicted jointly. For each single-target prediction scenario, the selected target variable was excluded from the predictor set, while all remaining variables were used as explanatory variables. Accordingly, when GDP was defined as the target variable, CO₂ emissions, Health Expenditure, and the remaining socio-economic indicators were included as predictors. The same approach was applied to the CO₂ emission and Health Expenditure prediction scenarios. This design was adopted to examine predictive relationships among interconnected economic, environmental, and health indicators rather than to infer causality.. During training, the dataset was split into 80% for training and 20% for testing. To evaluate prediction performance, Coefficient of Determination (R^2^), Root Mean Square Error (RMSE), and Mean Absolute Error (MAE) metrics were used. The analysis utilized the following algorithms: Extra Trees (ET), RF, HistGradientBoosting (HGB), GB, XGB, Decision Tree (DT), and Ridge Regression (RR), as well as a hybrid ensemble model (ET + HGB + XGB) combining some of these models. In the final stage, the performance of all models was compared, and a final interpretation and discussion were conducted. This study is designed as an association-based ML analysis using pooled country-year observations. The primary objective is to model predictive relationships among economic, environmental, and health indicators while comparing the performance of different machine learning algorithms. Therefore, the results should be interpreted as predictive associations rather than forecasts of future outcomes.

### Data source and scope

The dataset used in this study was compiled from the World Development Indicators (WDI) platform, an open-access database of the World Bank that contains economic, environmental, and health indicators for countries from 1960 to 2024. The dataset has a country-year panel structure, with annual observations for 217 countries. After data cleaning and preprocessing, the final dataset consists of 14,105 observations and 10 variables. The analysis includes the following variables: per capita CO₂ emissions, per capita GDP, per capita health expenditure, FDI inflows, trade openness, urbanization rate, patent applications, research and development (R&D) expenditures, natural resource rents, and goverment final consumption expenditures. The selected variables were chosen based on their frequent use in the literature examining the interactions among economic development, environmental sustainability, and public health. The predictor set includes economic (GDP, FDI, Trade, Government Consumption), technological (Patents, R&D Expenditure), environmental (CO₂ emissions, Natural Resource Rents), and demographic (Urban Population) indicators. Variables such as energy consumption and population aging, although frequently discussed in previous studies, were not included because consistent long-term observations for all countries and years were not available within the final analytical dataset. These indicators are among the basic macroeconomic and structural indicators widely used in the literature to explain economic growth, environmental sustainability, and Health Expenditures.

Due to missing or inconsistent observations in the raw dataset, a data preprocessing stage was performed before model development. Table 4 presents the study variables together with their descriptions, measurement units, data sources, and variable-specific missingness statistics. As shown in Table 4, the extent of missing data varied considerably across variables, with missingness rates ranging from 0.0% for Urban Population to 83.23% for R&D Expenditure. To preserve the country-year structure of the dataset and minimize information loss, missing values in the selected study variables were handled using median imputation. In this procedure, missing observations were replaced with the median value of the corresponding variable. Following data preprocessing, the final analytical dataset contained 14,105 country-year observations with no remaining missing values.

**Table 4:** Description of variables and missing data structure before data preprocessing

Variable	Description	Unit	Type	Source	Valid Observations Before Cleaning	Missing Observations	Missing Rate (%)	Final Observations
CO₂ Emissions	Per capita CO₂ emissions.	Metric tons per capita	Target	WDI	11,165	2,94	20.84	14,105
Health Expenditure	Public health expenditure per capita.	Current USD per capita	Target	WDI	4,344	9,761	69.20	14,105
FDI	Net inflows of FDI.	Current USD	Predictor	WDI	9,893	4,212	29.86	14,105
GDP	Gross domestic product per capita.	Current USD per capita	Target	WDI	11,339	2,766	19.61	14,105
Government Consumption	General government final consumption expenditure.	% of GDP	Predictor	WDI	8,487	5,618	39.83	14,105
Patents	Patent applications filed by residents.	Number	Predictor	WDI	3,856	10,249	72.66	14,105
R&D Expenditure	Research and development expenditure.	% of GDP	Predictor	WDI	2,365	11,74	83.23	14,105
Natural Resource Rents	Total natural resource rents.	% of GDP	Predictor	WDI	9,235	4,87	34.53	14,105
Trade Openness	Total trade as a percentage of GDP.	% of GDP	Predictor	WDI	8,819	5,286	37.48	14,105
Urban Population	Urban population share.	% of total population	Predictor	WDI	14,105	0	0.00	14,105

This approach was adopted to maintain dataset integrity and reduce the potential adverse effects of missing observations on model performance. To improve model performance and eliminate scale differences among variables, the data were standardized using the StandardScaler method. During the standardization process, each variable was transformed to have a mean of zero and a standard deviation of one. These preprocessing procedures ensured a consistent analytical framework for model training and enabled reliable performance comparisons across different machine learning algorithms.

**Fig. 2:**
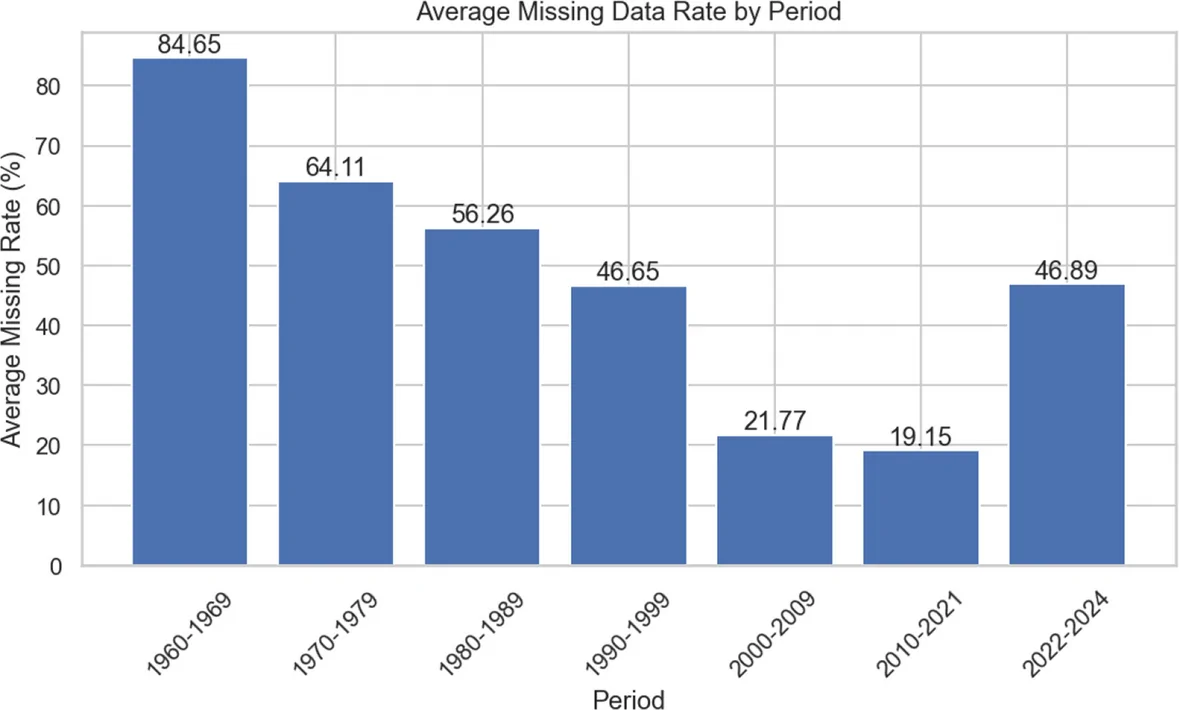
Temporal distribution of missing data rates in the WDI dataset (1960–2024)

Figure 2 further illustrates the temporal distribution of missing observations. Missingness was considerably higher in earlier decades and declined substantially after 2000, reflecting improvements in data availability and reporting practices. The relatively higher missingness observed in the most recent years (2022–2024) is likely associated with publication and reporting lags. These findings indicate that data availability is not uniformly distributed across time, which should be considered when interpreting the results. To mitigate excessive observation loss and preserve the longitudinal structure of the dataset, median imputation was applied during the preprocessing stage.

Although variables such as energy consumption and population aging are frequently discussed in the literature on environmental sustainability and health expenditures, they were not included in the present modeling framework because consistent and sufficiently complete long-term global observations for all 217 countries over the study period were not available. Instead, the analysis focused on a set of widely reported macroeconomic, environmental, technological, and demographic indicators available across countries and years. Environmental conditions were represented through per capita CO₂ emissions, which are commonly used as a proxy for environmental pressure in the sustainability literature.

Examination of the statistical distribution of the dataset revealed substantial variance differences across variables; therefore, feature scaling was applied. As a result of this process, a balanced final data matrix suitable for ML analysis was obtained. Table 5 presents the descriptive statistics for the variables used in the dataset. While the mean of the CO₂ emission variable is 101.06, its standard deviation is quite high (σ = 554.86), indicating substantial differences across countries. Similarly, in the per capita health expenditure variable, the low mean (438.61) relative to the standard deviation indicates a right-skewed distribution, with high-income countries contributing to greater variance. In the FDI inflow variable, the negative minimum value indicates that some countries experience capital outflows.

**Table 5:** Statistical information of the dataset

Variable	N	mean	SD	min	25%	50%	75%	max
CO₂ Emissions	14,105	101.061	554.864	0	1.478	7.239	31.107	13,124.73
Health Expenditure	14,105	438.612	715.457	0.259	276.027	276.027	276.027	8505.551
FDI	14,105	3.63E + 09	2.31E + 10	−3.4E + 11	30,027,879	1.25E + 08	4.87E + 08	7.34E + 11
GDP	14,105	10,832.61	17,819.44	122.679	1807.709	4002.556	10,522	247,170.2
General government consumption	14,105	15.949	6.421	1.788	13.849	15.294	16.692	147.735
Patents	14,105	3137.437	36,605.13	1	199.5	199.5	199.5	1,426,644
R&D Expenditure	14,105	0.637	0.423	0.005	0.574	0.574	0.574	6.019
Total natural resources rents	14,105	5.155	9.231	0	0.801	1.982	4.088	88.592
Trade	14,105	73.864	42.763	0.0210	56.776	66.686	77.555	863.195
Urban Population	14,105	52.039	25.743	1.839	31.206	51.739	72.179	100

In contrast, the very high maximum value suggests a high variance and an unbalanced distribution. The per capita income variable also varies widely, ranging from 122.67 to 247,170.2, revealing significant economic inequality between countries. The very high standard deviation of the patent number variable compared to the mean indicates that innovation production is concentrated in a small number of countries. While the R&D expenditure variable exhibits relatively low variance, the wide distribution of the natural resource rent variable shows a clear divergence in the dataset of resource-rich countries. When the trade ratio and urbanization variables are examined, medium-to-high variance but more balanced distributions are observed at the global level. In general, the high standard deviations across most variables indicate that the dataset has a heterogeneous structure. This provides an advantage for testing the generalization ability of ML models and reveals that the dataset comprises countries with diverse economic and structural profiles.

**Fig. 3:**
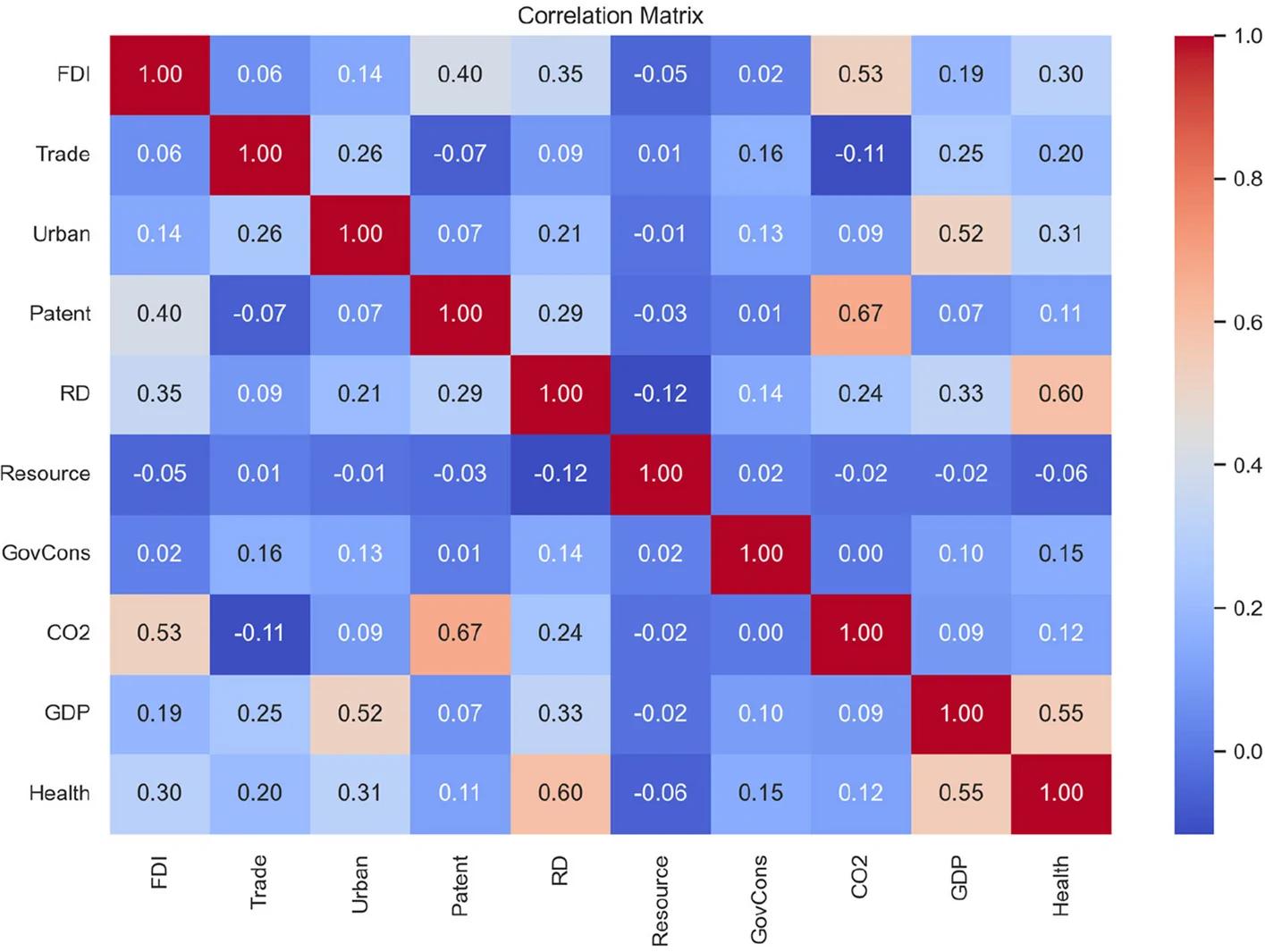
Correlation matrix of the study variables showing pairwise Pearson correlation coefficients among economic, environmental, technological, and health indicators

When the correlation matrix is examined, it is evident that there are relationships at different levels among the variables (Fig. 3). In particular, a strong positive relationship (*r* = 0.67) was found between the number of patents and CO₂ emissions, indicating that is associated with higher carbon emissions Similarly, the high correlation between R&D expenditures and health expenditures (*r* = 0.60) reveals that health investments also increase in countries with high innovation capacity. The positive relationship observed between per capita income, an indicator of economic well-being, and health expenditures (*r* = 0.55) supports the results of Rana et al. [61]. The moderate-to-high level relationship between urbanization rate and per capita income (*r* = 0.52) suggests that is associated with higher GDP levels.

The positive correlation between FDI and CO₂ emissions (*r* = 0.53) suggests that capital inflows is associated with higher CO₂ emission levels through industrial production activities. The weak negative relationship (*r* = − 0.11) between trade ratio and CO₂ emissions suggests that foreign trade may, in some cases, contribute to the spread of cleaner production technologies. In contrast, the lack of significant correlation between the natural resource rents variable and other variables suggests that it exhibits heterogeneous behavior across countries and may have limited explanatory power in forecasting models. Overall, the results reveal that structural factors such as economic growth, technology investments, and urbanization are associated with both environmental and health indicators, confirming that the selected attributes contain meaningful information for forecasting models.

### ML models

In this study, numerous ML algorithms with different learning paradigms were used to model the complex relationships between economic, environmental, and public health indicators and to improve the prediction accuracy of target variables. The main objective of the model selection process is to identify algorithms that are most suitable for the dataset structure by comparing methods with different learning mechanisms and statistical assumptions. Accordingly, models that can capture both linear and nonlinear relationships, work with high-dimensional data, are robust against outliers, and are widely used in the literature in regression problems were preferred.

The algorithms used in the study consist of seven main models: ET, RF, HGB, XGB, DT, and RR. The main reason for choosing these algorithms is that each represents a distinct learning approach and can model data patterns from different perspectives. For example, tree-based methods can automatically capture nonlinear relationships and variable interactions, boosting-based models iteratively optimize error minimization, and linear regression-based methods offer advantages in terms of parameter interpretability and model simplicity.

Decision tree-based methods are non-parametric models that create decision rules by branching the data based on feature values. A major advantage of these models is that they do not require strong distributional assumptions and can capture complex relationships through interpretable decision rules. However, since individual decision trees can exhibit high variance, an ensemble learning approach has been adopted in this study. RF and ET algorithms are bagging-based methods that combine predictions by creating multiple trees. These models reduce overtraining and improve generalization performance through random sampling and random feature selection. Boosting family algorithms, on the other hand, are based on the sequential training of weak learners. GB minimizes model errors via gradient-based optimization, while the HGB method reduces computational cost by discretizing continuous variables into histograms and provides faster training on large datasets.

The XGB algorithm, with its features such as regularization, parallel processing support, and advanced loss function optimization, is among the high-performance models in the literature. Therefore, the performance of boosting-based methods has been examined comparatively in this study. The RR algorithm, representing the linear model family, is a regularized version of multiple linear regression. This method reduces multicollinearity by limiting coefficient magnitudes and thereby increasing model stability. The purpose of including this model in the study is to compare the performance of complex algorithms with that of a simple yet powerful linear reference model. Four different prediction scenarios were created in the modeling process. The first three scenarios consist of regression problems with a single target variable and cover the prediction of CO₂ emissions, GDP, and Health Expenditure per capita variables, respectively. The fourth scenario is a multiple-output regression problem where these three variables are predicted simultaneously. To perform multiple-target prediction, the algorithms were extended using the MultiOutputRegressor, and an independent regression model was trained for each target variable. Thanks to this approach, the model’s ability to predict multiple dependent variables simultaneously was evaluated. To improve model performance and combine the strengths of different algorithms, a hybrid ensemble model, ET + HGB + XGB, was also designed. The proposed ensemble framework is based on the arithmetic averaging of predictions generated by three tree-based algorithms (ET, HGB, and XGB). Its purpose is not to introduce a novel ML architecture but to evaluate whether combining complementary algorithms can improve predictive performance and robustness across different prediction scenarios. In this hybrid approach, the prediction outputs of the ET, HGB, and XGB models are combined using the arithmetic mean method. Ensemble learning strategies are widely used in the literature because they can improve model stability and generalization performance by combining predictions from different algorithms. Hybrid models may provide performance advantages, particularly in heterogeneous datasets where a single algorithm may not perform consistently across different data patterns. All algorithms were trained using the same data preprocessing steps, and the same training-test data split was used to ensure fair comparison. All analyses were conducted in Python using the pandas, NumPy, Matplotlib, Seaborn, Scikit-learn, XGB, and Shapley Additive Explanations (SHAP) libraries. A fixed random seed was used for data splitting (random_state = 42). The dataset was divided into 80% training and 20% testing subsets using the train_test_split function. Feature scaling was performed after the train-test split: the StandardScaler was fitted only on the training data and then applied to the test data. This procedure was used to prevent information from the test set from influencing the scaling parameters. Hyperparameter optimization was not performed. Instead, the models were trained using predefined parameter settings, which are summarized in Table 6. Cross-validation was not applied in the main analysis, and model performance was evaluated using the held-out 20% test set. Missing values were handled using median imputation during data preprocessing to preserve the country-year structure of the dataset and minimize information loss. The final analytical dataset consisted of 14,105 country-year observations with no remaining missing values.

**Table 6:** Hyperparameter settings used in the study

Model	Hyperparameter settings
Extra Trees	n_estimators = 300, random_state = 42
Random Forest	n_estimators = 300, random_state = 42
HistGradientBoosting	sklearn default settings
XGB	n_estimators = 300, learning_rate = 0.05, max_depth = 6
Decision Tree	sklearn default settings
GB	sklearn default settings
Ridge Regression	alpha = 1.0
Hybrid ET + HGB + XGB	Arithmetic mean of ET, HGB, and XGB predictions; ET: n_estimators = 300; HGB: default parameters; XGB: n_estimators = 300

To quantitatively evaluate model performance, three key performance metrics—R^2^, RMSE, and MAE—were used. The R^2^ metric measures overall fit by showing the proportion of variance the model explains, while RMSE reflects the magnitude of the prediction error and is more sensitive to large errors. MAE is the average deviation of the model predictions, measured as the absolute mean error. When these three metrics are evaluated together, a comprehensive performance analysis can be conducted regarding model accuracy, error distribution, and generalization success. In conclusion, this study systematically compares numerous algorithms representing different learning strategies on the same dataset, aiming to determine the model best suited to the data structure. This approach not only identifies the model with the highest accuracy but also contributes methodologically to the literature by revealing which algorithmic class is more suitable for the problem structure.

### Theoretical justification of SHAP analysis

To enhance the interpretability of machine learning models and to better understand the predictive relationships among economic growth, environmental sustainability, innovation, and health expenditure indicators, this study employs SHAP, one of the most widely adopted XAI frameworks. SHAP is grounded in the Shapley value concept originally proposed in cooperative game theory by Shapley [62], where the total payoff generated by a coalition is fairly distributed among participating players according to their marginal contributions.

In the context of machine learning, explanatory variables are treated as players in a cooperative game, while the model prediction represents the total payoff. Accordingly, SHAP decomposes a prediction into additive feature-level contributions and quantifies the marginal effect of each variable on the model output. The theoretical formulation of SHAP is based on the unique Shapley solution, which satisfies the desirable properties of local accuracy, consistency, and missingness [63]. These properties ensure that feature attributions remain mathematically coherent and theoretically justified across different model structures. Unlike conventional feature importance measures that provide only global rankings of variables, SHAP enables both global and local interpretability. Global explanations identify the most influential predictors across the entire dataset, whereas local explanations reveal how individual variables contribute positively or negatively to a specific prediction. Therefore, SHAP offers a more comprehensive understanding of model behavior than traditional variable importance approaches.

Recent studies have emphasized that explainable artificial intelligence methods should not be viewed solely as post-hoc interpretation tools but also as mechanisms for uncovering hidden structural relationships within complex socioeconomic systems [64, 65]. In this perspective, SHAP provides an analytical bridge between predictive performance and theoretical interpretation by revealing how different variables jointly contribute to model outputs.

The use of SHAP in the present study is particularly appropriate because the relationships among GDP, CO₂ emissions, health expenditure, innovation, trade openness, and urbanization are inherently multidimensional and potentially nonlinear. While the ensemble machine learning models employed in this research achieve high predictive performance, their internal decision mechanisms are not directly observable. SHAP enables the decomposition of these black-box predictions into interpretable feature contributions, thereby facilitating a transparent evaluation of the variables that emerge as dominant predictors. Consequently, SHAP was adopted as the primary explainability framework to support both predictive interpretation and structural understanding of the associations identified by the machine learning models.

## Results

### Performance findings of ML models

**Fig. 4:**
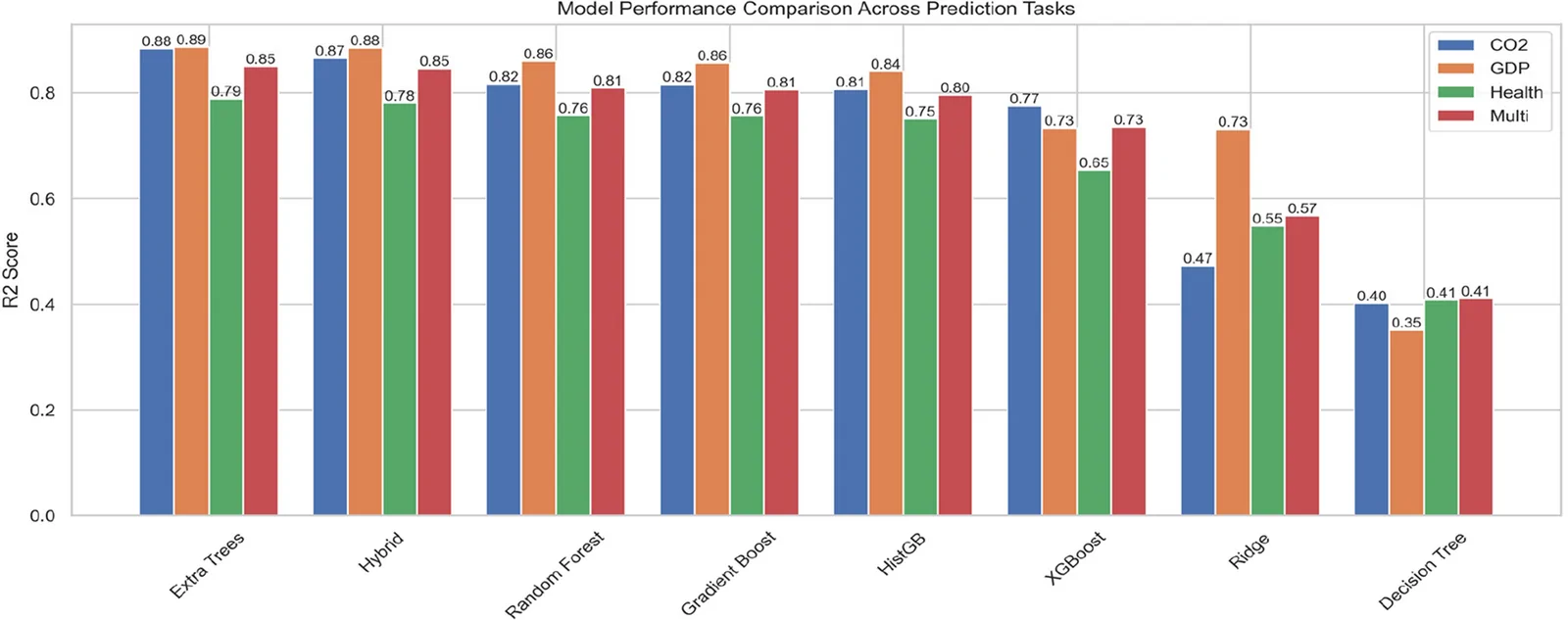
Comparison of R^2^ performance scores obtained from different machine learning algorithms across single-target (GDP, CO₂, Health Expenditure) and multi-target prediction scenarios

Figure 4 presents a comparative analysis of the R^2^ scores obtained by different ML algorithms across four prediction tasks (CO₂ emissions, GDP, health expenditure per capita, and multi-target prediction). When results are examined, it is found that tree-based ensemble methods generally achieve higher prediction accuracy than other models.

In terms of CO₂ emission prediction, the highest performance was obtained by the ET algorithm with an R^2^ value of 0.88. The hybrid model and HGB algorithms follow this method with values ​​of approximately 0.87 and 0.81, respectively. In contrast, the DT model achieved an R^2^ of only 0.40 on the same task, indicating that single tree structures can be insufficient for modeling complex data relationships. A similar trend was observed in GDP prediction. The ET model achieved the highest accuracy of approximately 0.89, while the Hybrid model showed strong performance at 0.88, and the RF model at approximately 0.86. The RR model maintained a moderate level of success at approximately 0.73, while the DT model showed the lowest performance at approximately 0.35. This reveals that linear models can only capture complex economic relationships to a limited extent. In health expenditure estimation, the performance of all models is relatively lower. The highest score belongs to the ET model with approximately 0.79, while other ensemble methods produced results in the range of 0.75–0.78. The XGB and RR models showed a significant decrease in performance on this task, with scores of approximately 0.65 and 0.55, respectively. This finding suggests that the health expenditure variable has a more complex and multi-factorial structure compared to other target variables.

In the multi-target estimation scenario, the most successful model was ET with a performance of around 0.85, while the hybrid model came in second with a result close to 0.85. GB and RF models showed similar performances, approximately 0.81. The fact that the DT model remained at only about 0.41 in the multiple prediction task reveals that the generalization ability of singular model structures is limited in multivariate prediction problems. When overall performance averages were evaluated, it was determined that the ET algorithm consistently produced the highest or near-highest results across all tasks. The hybrid model, by combining the strengths of different algorithms, achieved stable, high accuracy, emerging as the second most successful approach.

In contrast, the DT model performed the worst across all tasks. This result clearly shows that ensemble learning methods offer stronger generalization capabilities than single decision tree models. In conclusion, the findings show that ensemble-based ML algorithms are more effective in modeling complex nonlinear relationships between economic and environmental indicators. In particular, ET and hybrid model approaches have proven suitable for such multivariate datasets, providing high accuracy and stability in both single- and multi-objective prediction problems.

The R^2^ performance metrics presented in Fig. 3, as well as the RMSE and MAE metrics, are detailed in Table 7. When all results are evaluated, multi-target estimation achieves accuracy close to that of single models and is advantageous for practical use. Since the dataset contains complex nonlinear relationships, tree-based methods were the most suitable approach.

**Table 7:** Comparative performance of ML models for single and multi-target prediction

Model	Target	R^2^	RMSE	MAE
Extra Trees	Single (GDP)	**0.885932**	6378.071	2516.682
	Single (CO₂)	**0.883425**	144.5654	28.53498
	Single (Health)	0.780274	346.1364	89.87904
	Multi-target	**0.849877**	2289.591	878.3654
Hybrid	Single (GDP)	0.884901	6406.826	2957.379
	Single (CO₂)	0.865144	155.488	35.47271
	Single (Health)	**0.788657**	339.4694	103.7679
	Multi-target	0.845699	2322.135	1037.57
Random Forest	Single (GDP)	0.859479	7079.09	2878.619
	Single (CO₂)	0.816755	181.2499	35.11963
	Single (Health)	0.750675	368.7142	105.5197
	Multi-target	0.80897	2543.018	1006.419
XGB	Single (GDP)	0.856275	7159.348	3578.656
	Single (CO₂)	0.774464	201.0807	41.28101
	Single (Health)	0.756265	364.5575	121.3418
	Multi-target	0.795668	2574.995	1247.093
HistGB	Single (GDP)	0.840319	7546.303	3800.762
	Single (CO₂)	0.806092	186.4491	49.82107
	Single (Health)	0.756473	364.4014	130.0525
	Multi-target	0.805044	2691.904	1327.764
Decision Tree	Single (GDP)	0.733185	9754.668	3171.087
	Single (CO₂)	0.402029	327.4178	39.56127
	Single (Health)	0.547744	496.5909	127.1119
	Multi-target	0.567147	3308.654	1096.689
Gradient Boost	Single (GDP)	0.730661	9800.684	5207.889
	Single (CO₂)	0.815706	181.7682	52.18408
	Single (Health)	0.653706	434.5397	177.898
	Multi-target	0.734815	3469.398	1812.213
Ridge	Single (GDP)	0.351042	15,213.01	8540.346
	Single (CO₂)	0.473049	307.36	102.2797
	Single (Health)	0.408019	568.147	264.312
	Multi-target	0.410703	5362.84	2968.979

To further assess temporal robustness, the predictive performance of the proposed hybrid framework was evaluated across two broad periods characterized by relatively high data availability (2000–2012 and 2013–2024). As shown in Table 8, the model maintained strong predictive performance across both periods. The GDP prediction scenario achieved R^2^ values of 0.967 and 0.916, respectively, while the multi-target framework achieved R^2^ values of 0.942 and 0.893. Similarly, the Health Expenditure model produced high predictive accuracy in both periods, with R^2^ values exceeding 0.82.

**Table 8:** Period-based predictive performance (R^2^) of the proposed hybrid model

Period	CO2	GDP	Health	Multi-target
2000–2012	0.985 (*n* = 561)	0.967 (*n* = 561)	0.956 (*n* = 561)	0.942 (*n* = 1683)
2013–2024	0.759 (*n* = 516)	0.916 (*n* = 516)	0.824 (*n* = 516)	0.893 (*n* = 1548)

A moderate decline in predictive performance was observed during the 2013–2024 period, particularly for the CO₂ emissions model. This reduction may be associated with the increased proportion of missing observations and reporting delays observed in the most recent years of the dataset. Nevertheless, the overall results indicate that the proposed framework maintains stable predictive capability across different temporal subsets of the data. These findings provide additional evidence that the reported model performance is not driven solely by a specific historical period. Table 8, n represents the number of country-year observations from the test subset within each period used to calculate the reported performance metrics.

### XAI and variable interaction analysis

In this study, SHAP, one of the XAI techniques, was used to make the prediction mechanisms of ML models transparent and to determine the marginal relationship of variables on the dependent variable. Unlike traditional importance rankings, SHAP analysis quantitatively reveals the positive or negative contribution of variables to the prediction value for each observation unit.

**Fig. 5:**
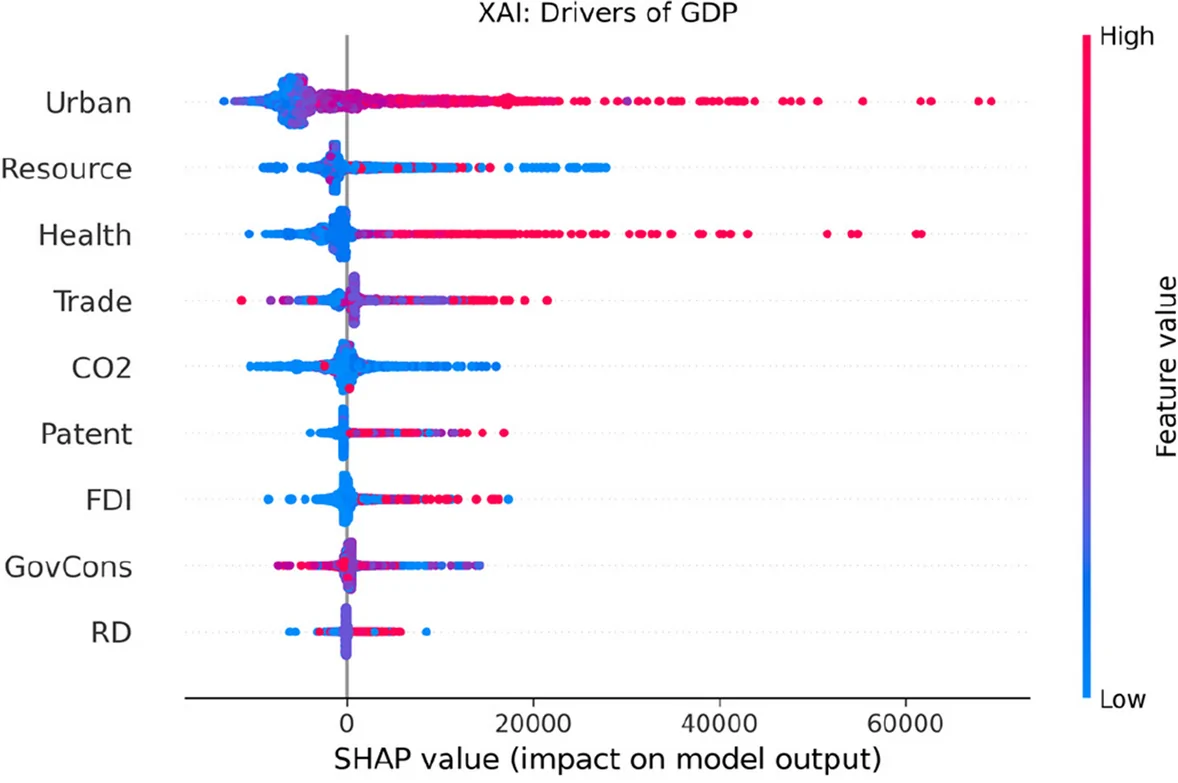
SHAP summary plot for GDP prediction, illustrating the relative predictive contributions of explanatory variables and their associations with model-predicted GDP values

When examining the SHAP summary graph of the GDP prediction model presented in Fig. 5, it is seen that the variables with the highest predictive contribution to GDP predictions are Trade and Urban. The concentration of red dots, representing high trade volume and urbanization rates, in the positive SHAP region confirms that these variables are strongly associated with higher model-predicted GDP values. The high ranking of Health and RD variables show substantial predictive importance in the GDP prediction model. The positive association between carbon emissions and GDP indicates that economic activities in the examined sample group continue to occur at the expense of environmental pollution (carbon-intensive growth). The strong contribution of urbanization may reflect the concentration of labor, infrastructure, and economic activities in urban areas, which are generally associated with higher productivity levels. Similarly, the importance of health expenditure suggests that investments in human capital and healthcare systems are closely linked to economic performance. The contribution of R&D activities further highlights the role of innovation capacity and technological development in supporting economic growth. Taken together, these findings suggest that GDP is associated not only with trade and production-related factors but also with broader dimensions of human capital, urban development, and innovation ecosystems.

**Fig. 6:**
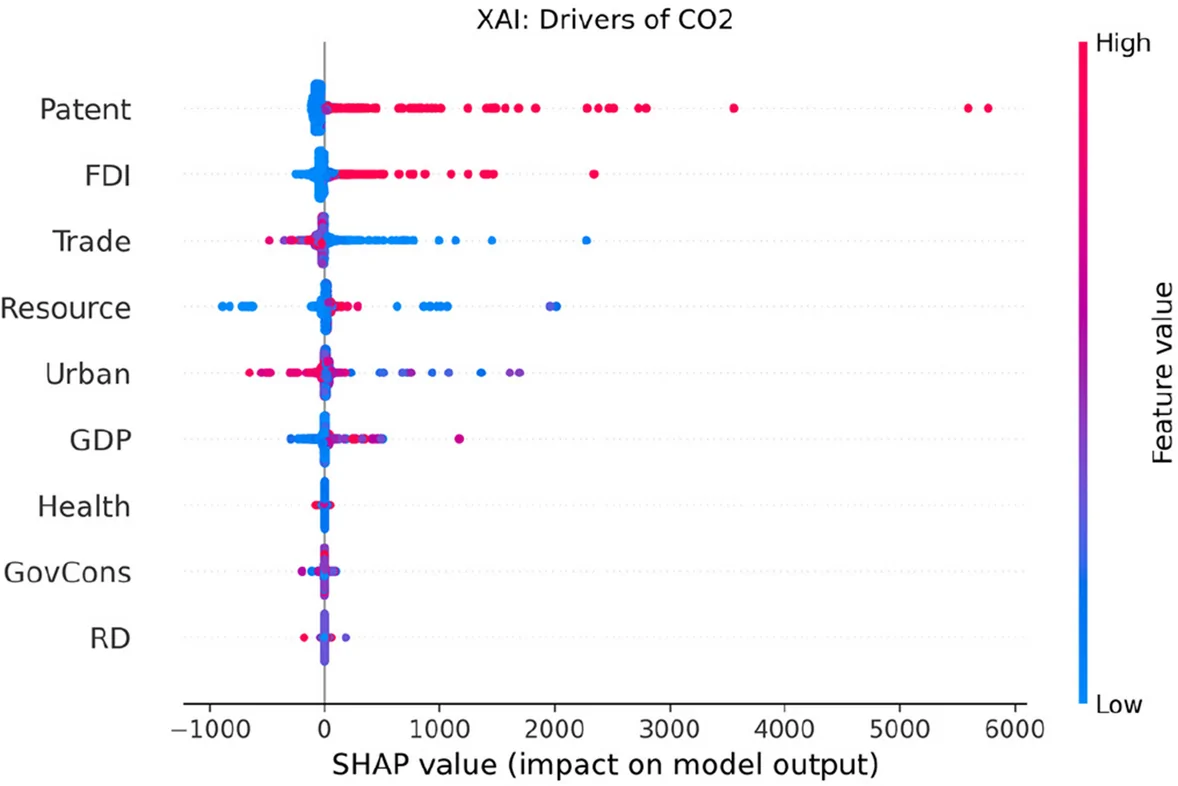
SHAP summary plot for CO₂ emission prediction, showing the relative predictive contributions of explanatory variables and their associations with model-predicted CO₂ emissions

The SHAP analysis focusing on CO₂ emissions (Fig. 6) identifies the variables with the greatest predictive contribution to the CO₂ prediction model. GDP exhibits the largest SHAP contribution, indicating a strong association between GDP levels and model-predicted CO₂ emissions. This finding may be related to increasing energy demand, transportation intensity, and infrastructure expansion in highly urbanized regions. In contrast, the contribution of R&D expenditures appears more heterogeneous across observations. The presence of negative SHAP values for R&D expenditures in some cases suggests that higher levels of technological development are associated with lower model-predicted emissions within certain observations of the dataset. Furthermore, patent-related indicators and FDI also emerge as important predictors of CO₂ emissions. Patent activity may reflect either industrial expansion associated with increased production and emissions or the development of cleaner technologies that support environmental sustainability. Similarly, FDI can contribute to higher emissions through expanded industrial activity while simultaneously facilitating technology transfer and the adoption of more efficient production systems. Overall, these findings highlight the complex and potentially nonlinear relationships between technological development, investment patterns, urbanization, and environmental sustainability.

**Fig. 7:**
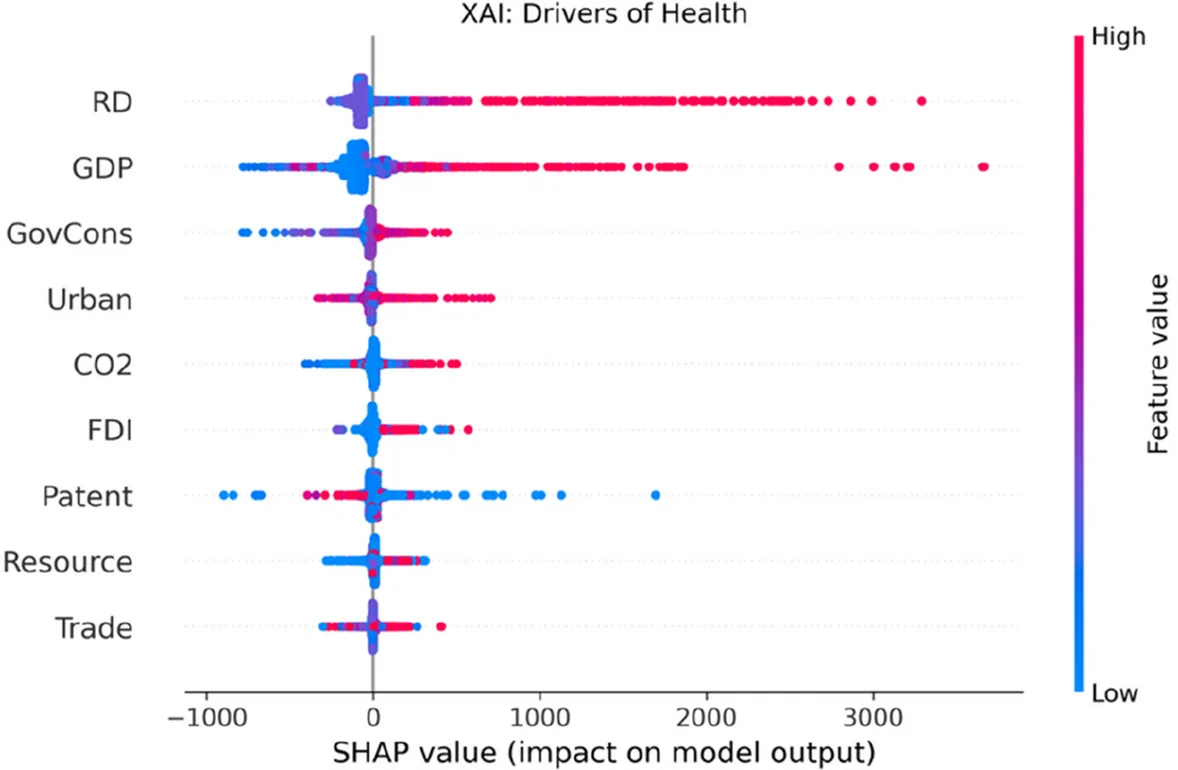
SHAP summary plot for health expenditure prediction, presenting the relative predictive contributions of explanatory variables and their associations with model-predicted health expenditure levels

The SHAP results for the model designed to estimate health expenditures (Fig. 7) highlight the variables with the greatest predictive contribution to health expenditure predictions. GDP has the highest predictive contribution in the health expenditure model and is strongly associated with predicted health expenditure levels. Government consumption (GovCons) also exhibits substantial predictive importance, indicating that it contributes meaningfully to model predictions of health expenditure. Furthermore, CO₂ emissions show a positive contribution in a number of observations, suggesting that higher CO₂ levels are associated with higher predicted health expenditures within the analyzed dataset. In addition, R&D expenditure emerges as one of the most influential predictors in the health expenditure model. This finding may reflect the tendency of innovation-oriented economies to invest more heavily in healthcare systems, medical technologies, and health-related research activities. The importance of GDP further suggests that economically developed countries generally possess greater financial capacity to allocate resources to healthcare services and infrastructure. Similarly, the contribution of government consumption may indicate the role of public sector capacity in supporting healthcare provision and social welfare systems. The positive contribution of CO₂ emissions observed in several cases may be associated with the health burden and healthcare costs arising from environmental degradation and pollution-related diseases. However, it should be emphasized that SHAP values quantify predictive contributions within the machine learning model and do not imply causal relationships among the variables. Therefore, these findings should be interpreted as model-based predictive associations rather than evidence of causal effects.

## Discussion

This study presents an empirical ML framework for analyzing the nonlinear interactions among macroeconomic variables, environmental factors, and health indicators. The analysis process is structured around two strategic focus points: the predictive associations between health and macroeconomic variables and GDP predictions, and the role of these variables in environmental pollution. The findings demonstrate that the dynamics between GDP, CO₂ emissions, and health expenditures require predictive approaches capable of capturing complex nonlinear relationships beyond the assumptions of traditional econometric models. The practical contribution of the study lies in demonstrating that a simple ensemble combination of ET, HGB, and XGB models can provide stable and competitive predictive performance across multiple sustainability-related prediction tasks. While Caravaggio et al. [60] highlighted the success of singular GB models, the proposed ‘Arithmetic Mean Based Ensemble Learning’ approach demonstrated a much more robust predictive performance against outliers by minimizing model variance in heterogeneous global datasets. The results of the multi-target modeling indicate that health expenditures exhibit substantial predictive importance within the GDP prediction models and are strongly associated with economic performance indicators. These findings do not imply that health expenditures cause changes in GDP levels; rather, they indicate that health expenditure variables contain information that is useful for predicting GDP within the analyzed dataset. This observation is broadly consistent with the associations reported by Zhang and Zhang [5] between health-related expenditures and economic outcomes. The observed predictive relationships are broadly consistent with previous studies that reported associations between health expenditures, environmental indicators, and economic performance [5, 54]. However, the present findings should be interpreted as model-based predictive associations rather than evidence of causal mechanisms. The SHAP analysis outputs are broadly consistent with findings reported by Yang et al. [66] and Abdelsamiea et al. [59], highlighting the predictive importance of demographic and socio-economic indicators within the analyzed dataset. Nevertheless, these results reflect model-attributed relationships rather than causal effects. Therefore, the reported SHAP importance values should be interpreted as indicators of predictive contribution within the trained models rather than evidence of directional or policy-relevant effects. The findings should be interpreted as predictive associations identified from historical country-year observations rather than as forecasts of future country-level outcomes. Consequently, the reported model performances reflect the ability of ML algorithms to capture patterns within the analyzed dataset.

In conclusion, the findings highlight predictive associations among health expenditures, economic growth, and environmental indicators across a large international dataset. These associations are broadly consistent with themes discussed in the sustainability and environmental economics literature, including the frameworks proposed by Shakoor and Ahmed [67] and Teixeira et al. [68]. While the present study does not establish causal mechanisms, it demonstrates the usefulness of ML approaches for identifying complex nonlinear patterns among economic, environmental, and health-related variables.

The findings may provide a useful reference for future studies investigating the interactions among economic, environmental, and public health indicators using predictive modeling approaches. It should be emphasized that the present study focuses on predictive modeling and variable importance analysis, and the reported relationships should not be interpreted as evidence of causal mechanisms.

## Conclusion

This research, based on the dynamic interactions among economic growth, environmental sustainability, and public health indicators, has developed a ML-based analytical framework for examining predictive relationships among economic, environmental, and health indicators.The ET + HGB + XGB hybrid ensemble model achieved competitive performance across the evaluated prediction scenarios. The model’s R^2^ value of 0.885 in GDP prediction and its performance in health expenditure and CO₂ emission prediction tasks demonstrate the ability of ML methods to capture complex nonlinear patterns in large-scale global datasets. The SHAP analysis indicates that variables such as patent activity and foreign direct investment showed relatively high predictive contributions in several model scenarios. These contributions should be interpreted as model-based predictive importance measures rather than estimates of causal effects. Since the analysis is based on pooled country-year observations, the results should not be interpreted as out-of-sample forecasts of future economic, environmental, or health outcomes.

An additional temporal robustness analysis was conducted to evaluate model performance across different post-2000 periods characterized by relatively high data availability. The results demonstrated that the proposed hybrid framework maintained strong predictive performance in both 2000–2012 and 2013–2024 periods. For GDP prediction, R^2^ values were 0.967 and 0.916, respectively, while the multi-target framework achieved R^2^ values of 0.942 and 0.893. Similarly, the Health Expenditure prediction model maintained high predictive accuracy across both periods (*R*^2^ = 0.956 and 0.824). Although a moderate decline in performance was observed during the 2013–2024 period, particularly for CO₂ emissions prediction, the results indicate that the predictive capability of the proposed framework remains relatively stable across different temporal subsets of the dataset. Overall, the findings highlight the usefulness of machine learning approaches for exploring predictive relationships among sustainability-related indicators and provide a basis for future studies incorporating additional variables, alternative validation strategies, and subgroup analyses.

### Limitations and suggestions for future research

The main limitation of this study is that, although it is based on a massive dataset covering 217 countries, it cannot analyze short-term dynamics of the variables due to the use of annual macroeconomic data. In addition, the analysis is based on pooled country-year observations and employs a random train-test splitting strategy. Although an additional period-based robustness analysis demonstrated stable predictive performance across post-2000 periods, future studies may further evaluate model robustness using stricter temporal or country-level validation procedures. Future studies may further evaluate the robustness of ML models using time-based validation, country-level grouped validation, and regional or income-group-specific analyses. Another limitation of the present study is that all country-year observations were analyzed within a pooled global framework. Potential regional, income-group, or development-level heterogeneity was not explicitly examined. Future studies may investigate whether the observed predictive relationships differ across developed and developing countries or among alternative regional groupings. Another limitation of this study concerns the interpretation of SHAP-based findings. Although SHAP values provide valuable insights into the internal behavior of machine learning models by quantifying the predictive contribution of individual variables, they do not establish causal relationships among variables. Therefore, the associations identified between economic growth, health expenditure, environmental sustainability indicators, and other explanatory variables should be interpreted as model-based predictive relationships rather than evidence of causal effects. Consequently, policy implications derived from these findings should be considered with appropriate caution.

Future studies may extend the proposed framework by incorporating temporal modeling approaches, such as LSTM-based architectures, together with sectoral, regional, or other disaggregated datasets. Future studies may extend the proposed framework by incorporating energy consumption, age-structure indicators, and additional environmental variables where data availability permits.

## Data Availability

Availability of data and materials: Availability of data and materials: All data used in this study were obtained from the publicly available World Bank WDI database. Data were extracted in January 2026. The WDI variables used in the analysis are given in Table 4, and the data preprocessing and modeling procedures are described in detail in the Methods section. The processed dataset and analysis workflow are available from the corresponding author upon reasonable request.

## Abbreviations

ANN: Artificial Neural Networks
BRICS: Brazil, Russia, India, China, and South Africa
CO₂: Carbon Dioxide
DT: Decision Tree
EKC: Environmental Kuznets Curve
ET: Extra Trees
FDI: Foreign Direct Investment
GB: Gradient Boosting
GDP: Gross Domestic Product
GovCons: General Government Final Consumption Expenditure
HGB: Histogram-based Gradient Boosting
LSTM: Long Short-Term Memory
MAE: Mean Absolute Error
ML: Machine Learning
NOX: Nitrogen Oxides
OECD: Organisation for Economic Co-operation and Development
R&D: Research and Development
RF: Random Forest
RMSE: Root Mean Square Error
RR: Ridge Regression
SHAP: Shapley Additive Explanations
SOM: Self-Organizing Maps
SVR: Support Vector Regression
WDI: World Development Indicators
XAI: Explainable Artificial Intelligence
XGB: Extreme Gradient Boosting
